# Thrombomodulin/activated protein C system in septic disseminated intravascular coagulation

**DOI:** 10.1186/s40560-014-0050-7

**Published:** 2015-01-07

**Authors:** Takayuki Ikezoe

**Affiliations:** Department of Hematology and Respiratory Medicine, Kochi University, Nankoku, Kochi, 783-8505 Japan

**Keywords:** Thrombomodulin, Activated protein C, Disseminated intravascular coagulation, Sepsis

## Abstract

The thrombomodulin (TM)/activated protein C (APC) system plays an important role in maintaining the homeostasis of thrombosis and hemostasis and maintaining vascular integrity *in vivo*. TM expressed on vascular endothelium binds to thrombin, forming a 1:1 complex and acts as an anticoagulant. In addition, the thrombin-TM complex activates protein C to produce APC, which inactivates factors VIIIa and Va in the presence of protein S, thereby inhibiting further thrombin formation. Intriguingly, APC possesses anti-inflammatory as well as cytoprotective activities. Moreover, the extracellular domain of TM also possesses APC-independent anti-inflammatory and cytoprotective activities. Of note, the TM/APC system is compromised in disseminated intravascular coagulation (DIC) caused by sepsis due to various mechanisms, including cleavage of cell-surface TM by exaggerated cytokines and proteases produced by activated inflammatory cells. Thus, it is reasonable to assume that reconstitution of the TM/APC system by recombinant proteins would alleviate sepsis and DIC. On the basis of the success of the Protein C Worldwide Evaluation in Severe Sepsis (PROWESS) trial, the FDA approved the use of recombinant human APC (rhAPC) for severe sepsis patients in 2002. However, subsequent clinical trials failed to show clinical benefits for rhAPC, and an increased incidence of hemorrhage-related adverse events was noted, which prompted the industry to withdraw rhAPC from the market. On the other hand, recombinant human soluble TM (rTM) has been used for treatment of individuals with DIC since 2008 in Japan, and a phase III clinical trial evaluating the efficacy of rTM in severe sepsis patients with coagulopathy is now ongoing in the USA, South America, Asia, Australia, European Union, and other countries. This review article discusses the molecular mechanisms by which the TM/APC system produces anticoagulant as well as anti-inflammatory and cytoprotective activities in septic DIC patients.

## Introduction

Disseminated intravascular coagulation (DIC) is characterized by the systemic activation of coagulation caused by various underlying diseases, with sepsis being the leading cause [1]. The initial step of hypercoagulability caused by sepsis is triggered by tissue factor (TF) whose expression is induced on cell surfaces of vascular endothelial cells and innate immune cells by pathogen-associated molecular patterns (PAMPs), such as lipopolysaccharide (LPS) and peptidoglycan [2-5]. TF forms a complex with activated factor VII (FVIIa), which orchestrates the coagulation pathway and generates thrombin [2-5]. Thrombin converts fibrinogen to fibrin and causes fibrin deposition in systemic microvasculature in concert with FXIIIa, which facilitates crosslinking of fibrin monomers [6]. In addition to PAMPs, alarmins such as high-mobility group box 1 protein (HMGB1) and damage-associated molecular patterns (DAMPs), and nuclear architectural chromatin-binding proteins including histones also activate inflammation and coagulation after being liberated from necrotic/apoptotic cells or activated by inflammatory cells [7-9]. For example, HMGB1, released into circulation, binds to receptor for advanced glycation end products (RAGE) on endothelial and inflammatory cells and stimulates the production of cytokines such as interleukin-6 (IL-6) and tumor necrosis factor α (TNFα), which further activate systemic inflammation and hypercoagulability [10]. Histones, especially histones H3 and H4, stimulate the production of inflammatory cytokines including TNFα and IL-6 via toll-like receptors 2 and 4, which trigger activation of coagulation pathways [11]. Recent studies have found that neutrophil extracellular traps (NETs), extracellular DNA fibers comprising histone and neutrophil antimicrobial proteins released in response to microbial stimuli, also stimulate platelets and coagulation [12-14].

In physiological conditions, the anticoagulant system comprising antithrombin (AT), thrombomodulin (TM)/activated protein C (APC), and tissue factor pathway inhibitor (TFPI) is activated in response to hypercoagulability [3,4]. However, this anticoagulant system is severely compromised in individuals with septic DIC by various mechanisms; for example, levels of AT are decreased in septic patients due to rapid clearance from circulation after forming a complex with thrombin or its degradation by elastases released from activated neutrophils [15]. TM on the surfaces of vascular endothelial cells is cleaved by neutrophil elastases. In addition, the expression of TM in endothelium is downregulated by inflammatory cytokines including TNFα [16]. Secondary fibrinolysis is also compromised in septic DIC patients, mainly because of an increase in the expression of plasminogen activator inhibitor-1 (PAI-1) in vascular endothelial cells that is mediated by endotoxin and TNFα [17]. Thus, exaggerated coagulation in parallel with impaired anticoagulant and fibrinolysis systems result in continuous thrombus formation in systemic small and midsize vessels, leading to organ dysfunction, a clinical feature of septic DIC. In addition, the exhaustion of coagulation factors and platelets results in hemorrhage.

Recombinant human soluble TM (rTM) comprises the extracellular domain of TM and has been used for treatment of DIC since 2008 in Japan [18,19]. Post-marketing surveillance has proven the effectiveness and safety of this new treatment strategy to reconstitute the TM/APC system for the management of DIC in both pediatric and adult patients [20,21]. In addition, increasing numbers of retrospective studies and case reports suggest that the anti-inflammatory and cytoprotective actions of rTM are effective in managing DIC caused by various underlying diseases, including sepsis and fetal complications developed after hematopoietic stem cell transplantation [22-28]. rTM is now under the spotlight, and a phase III clinical trial evaluating its efficacy in severe sepsis patients with coagulopathy is now ongoing in the USA, South America, Asia, Australia, European Union, and other countries. (https://clinicaltrials.gov/ct2/show/NCT01598831?term=ART-123&rank=2). The use of recombinant APC was also shown effective in reducing mortality in severely ill sepsis patients as shown in the Protein C Worldwide Evaluation in Severe Sepsis (PROWESS) trial and has been recommended for patients with severe sepsis and DIC by the British Committee for Standards in Haematology (BCSH) [29,30]. However, subsequent clinical trials, including the industry-sponsored (Eli Lilly, Indianapolis, IN, USA) PROWESS-SHOCK trial, failed to show a benefit from the use of rhAPC in severe sepsis patients (http://www.ema.europa.eu/docs/en_GB/document_library/Press_release/2011/10/WC500116970.pdf). Given the industry's decision to withdraw rhAPC from the market, the BCSH withdrew their recommendation of rhAPC in patients with severe sepsis and DIC [31]. Nonetheless, accumulated results obtained from *in vitro* and *in vivo* preclinical studies support the efficacy of APC in septic DIC, which has attracted the attention of physicians. This review will focus on the roles of the TM/APC system in coagulation, inflammation, and cytoprotection.

## Review

### Anticoagulant function of TM/APC

**Figure 1:**
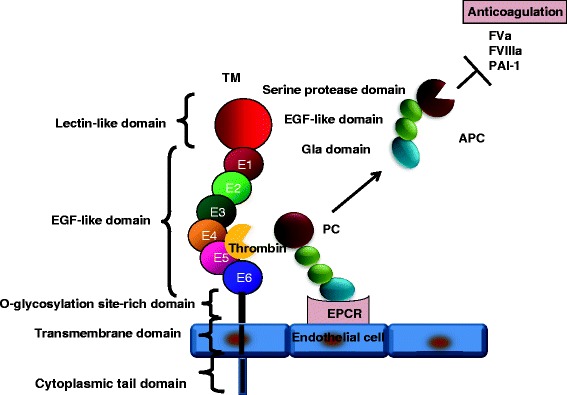
**Anticoagulant function of TM/APC.** TM, thrombomodulin; PC, protein C; APC, activated PC; EPCR, endothelial cell protein C receptor; PAI-1, plasminogen activator inhibitor-1; EGF, epidermal growth factor; Gla, gamma-carboxyglutamic acid.

TM is a glycosylated type I transmembrane molecule of 557 amino acids with multiple domains. Each domain possesses distinct properties. The molecule consists of an NH_2_-terminal lectin-like region followed by six tandem epidermal growth factor (EGF)-like structures, an O-glycosylation site-rich domain, a transmembrane domain, and a cytoplasmic tail domain (Figure 1) [32]. TM is ubiquitously expressed on endothelial cells and binds to thrombin, forming a 1:1 complex via the fourth and fifth (E45) repeats in an EGF-like domain and acting as an anticoagulant [33]. In addition, the thrombin-TM complex activates protein C (PC), a vitamin K-dependent anticoagulant serine protease, to produce APC [34,35]. APC is composed of four domains: an amino-terminal gamma-carboxyglutamic acid (Gla) domain, two epidermal growth factor-like regions, and an enzymatic serine protease domain (Figure 1) [36]. APC inactivates FVIIIa and FVa by cleavage of these coagulant factors at Arg336 and Arg562 or at Arg306 and Arg506, respectively, in the presence of protein S, thereby inhibiting further thrombin formation (Table 1) [37,38]. The minimum structure of TM essential to generate APC is localized in E456 repeats of the EGF-like domain [39]. Endothelial cell protein C receptor (EPCR) expressed on the cell surfaces of endothelium markedly facilitates the generation of APC by binding the Gla domain of PC and presenting it to the thrombin/TM complex [40].

**Table 1:** **Roles of TM/APC in septic DIC**

	**Functions**	**References**
Lectin-like domain of TM	Inhibits HMGB1 (alarmins)	[63]
	Inhibits LPS (PAMPs)	[64]
	Inhibits adhesion of neutrophils to endothelial cells	[65]
	Inhibits complement factors	[70]
	Binds to thrombin	[33]
EGF-like domain of TM	Binds to thrombin	[33]
	Generates APC	[34,35,39]
	Protects vascular endothelial cells	[72]
	Inhibits complement factors	[68,69]
	Inhibits coagulation	[37,38]
	Enhances fibrinolysis by inhibition of PAI-1	[41]
APC	Activates G protein-coupled receptor and protects vascular endothelial cells	[43-47], [48,49]
	Increases Tie-2 and Ang1, and protects vascular endothelial cells	[51-54]
	Mitigates acute lung injury	[56]
	Binds to ApoER2 and activates prosurvival signals	[57,58]
	Binds to integrin receptors and inhibits inflammation	[59]
	Inhibits inflammation via inactivation of NF-κB	[60,61]
	Cleaves histones (DAMPs)	

DIC, disseminated intravascular coagulation; TM, thrombomodulin; APC, activated protein C; HMGB1, high-mobility group box 1; PAMPs, pathogen-associated molecular patterns; DAMPs, damage-associated molecular patterns; PAI-1, plasminogen activator inhibitor-1; Tie2, tyrosine kinase with Ig-like loops and epidermal growth factor homology domains-2, Ang1, angiopoietin 1; ApoER2, apolipoprotein E receptor 2, NF-κB, nuclear factor-κ B.

In addition to its anticoagulant activity, APC enhances fibrinolysis by inactivation of PAI-1 (Table 1) [41]. Previous studies have shown that APC inhibits PAI-1 and promotes thrombolysis in individuals with acute myocardial infarction [42].

### Anti-inflammatory and cytoprotective functions of APC

**Figure 2:**
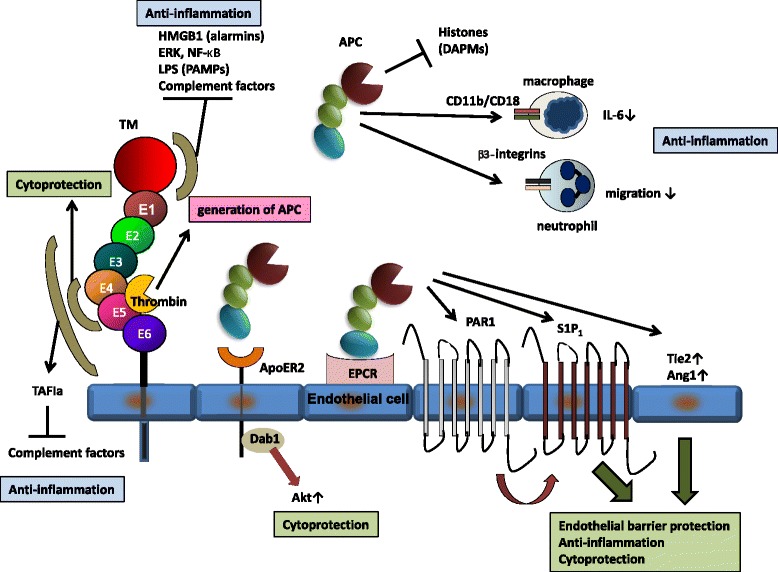
**Anti-inflammatory and cytoprotective functions of TM/APC.** HMGB1, high-mobility group box 1; PAR1, protease-activated receptor-1; S1P_1_, sphingosine 1-phosphate receptor; TAFIa, active thrombin-activatable fibrinolysis inhibitor; EPCR, endothelial cell protein C receptor; ERK, extracellular signal-regulated kinase; NF-κB, nuclear factor-κ B; Ang1, angiopoietin 1; Tie2, tyrosine kinase with Ig-like loops and epidermal growth factor homology domains-2, ApoER2, apolipoprotein E receptor 2; Dab1, disabled-1; PAMPs, pathogen-associated molecular patterns; DAMPs, damage-associated molecular patterns.

APC cleaves G protein-coupled receptor protease-activated receptor-1 (PAR1) at Arg46 and mediates a downstream signal transduction pathway, leading to the generation of anti-inflammatory as well as cytoprotective activities (Table 1) [43]. Formation of the binding complex with its receptor EPCR is essential for APC to activate PAR1 signaling; an EPCR mutant (EPCR A154) lacking the binding ability to APC fails to activate PAR1 [44]. Anti-EPCR antibodies block the ability of APC to activate the PAR1-mediated prosurvival signal transduction pathways [45]. PAR1 requires another G protein-coupled receptor, sphingosine 1-phosphate receptor (S1P_1_), to mediate the action of APC to enhance endothelial barrier protection (Figure 2) [46]. S1P_1_ activates phosphoinositide 3-kinase (PI3K) and mitogen-activated protein kinase (MAPK), including extracellular signal-regulated kinase (ERK), leading to endothelial barrier protection and angiogenesis [45,47]. The endothelial cell-specific tyrosine kinase receptor Tie2 also plays a role in APC-mediated cytoprotection; APC increases levels of Tie2 and its ligand angiopoietin 1 (Ang1) in concordance with the upregulation of the tight junction protein zona occludens (ZO)-1 in human umbilical vein endothelial cells (HUVECs) (Table 1, Figure 2) [48,49].

The cytoprotective function of APC is not limited to vascular endothelial cells. APC blocks staurosporin-mediated apoptosis of neuronal cells in association with the inhibition of caspase 8, the nuclear translocation of apoptosis-inducing factor, and the induction of p53. In addition to PAR1, PAR3 is involved in APC-mediated neuronal cell protection [50]. The cytoprotective function of APC has also been shown in an acute lung injury (ALI) model. APC protects alveolar epithelial barrier function via EPCR/PAR1/S1P_1_-dependent mechanisms in concert with the inhibition of the small GTPase RhoA and the activation of Rac1, which inhibits formation of actin-myosin stress fibers [51]. Inhaled aerosolized rhAPC attenuates ALI in endotoxin-induced acute respiratory distress syndrome (ARDS) and in a ventilation-induced lung injury murine model [52,53]. Curiously, prophylactic use of a cytoprotective-selective mutant form of APC with reduced anticoagulant activity attenuates *Pseudomonas aeruginosa*-induced ALI in a murine model and significantly prolongs survival of these mice compared with control vehicle-treated mice (Table 1) [51,54].

Intriguingly, APC rescues mice from radiation-induced bone marrow failure [55]. APC's mitigation of radiation toxicity is not merely dependent on its effect on hematopoietic cells, as augmented TM/APC signaling in hematopoietic progenitor cells is not able to stimulate their colony-forming ability *in vitro*. A series of experiments utilizing mutant forms of APC, including the cytoprotective-selective 5A APC variant with reduced anticoagulant activity and the anticoagulant-selective Glu149Ala variant lacking anti-apoptotic activity, suggest that radiomitigation by APC is independent of EPCR/PAR1 but is preserved in the Glu149Ala variant [55]. The precise mechanisms by which the APC Glu149Ala variant supports hematopoiesis in a radiation-induced bone marrow failure murine model remain unknown.

In addition to EPCR, APC binds to apolipoprotein E receptor 2 (ApoER2) in U937 monocytic leukemia cells. Upon binding to ApoE2R2, APC causes phosphorylation of Tyr220 in adaptor protein disabled-1 (Dab1), Ser473 in Akt, and Ser9 in glycogen synthase kinase 3β (GSK3β), which is dependent on PI3K but not on EPCR/PAR1 signaling [56]. ApoER2-mediated activation of this prosurvival signaling may contribute to the cytoprotective function of APC (Table 1, Figure 2).

Another binding partner of APC concerns the integrin family; APC binds to the heterodimeric integrin receptor CD11b/CD18 within specialized membrane microdomains/lipid rafts and activates PAR1/S1P_1_ signaling, resulting in the suppression of inflammatory responses in LPS-activated macrophages (Table 1, Figure 2) [57]. Another integrin class that elicits anti-inflammatory functions of APC is the β_1_/β_3_-integrins. APC binds to β_1_/β_3_-integrins and inhibits neutrophil migration in which the Arg-Gly-Asp (RGD) sequence plays a critical role (Table 1, Figure 2). The RGD peptide recapitulates the beneficial effects of rhAPC on survival in an LPS-challenged murine sepsis model [58].

The anti-inflammatory function of APC has been elegantly demonstrated by microarray analyses, which have found that APC alters the expression of various types of genes involved in inflammation, apoptosis, and cell adhesion in HUVECs. These include the anti-apoptotic Bcl-2, intracellular adhesion molecule 1, vascular cell adhesion molecule 1, and E-selectin [59]. APC inhibits the nuclear factor-κ B (NF-κB), a crucial transcription factor regulating the expression of genes involved in inflammation and cell survival, in endothelial cells. Modulation of NF-κB by APC at least in part plays a role in altered gene expression mediated by APC (Table 1) [59].

Intriguingly, APC binds to DAMPs, histone H3, and H4 through its densely anionic N-terminal Gla domain by means of electrostatic forces and subsequently cleaves these histones in a PAR1-independent manner (Table 1, Figure 2) [60,61]. Exposure of human umbilical vein EA.hy926 cells to histones causes cell toxicity, which is effectively blocked when these cells are cultured in the presence of histones together with APC, suggesting cytoprotective roles of APC against histones [60]. In addition, the injection of histones into mice causes their death within 1 hour in association with a massive accumulation of neutrophils in the alveolar microvasculature, a sign of augmented inflammation. Notably, concomitant use of APC rescues all mice challenged with a lethal dose of histones [60]. This anti-histone activity may be one of the most attractive functions of APC to rescue septic DIC patients, as significantly higher plasma levels of histone H3 are noted in non-survivors with septic DIC compared with survivors [62].

### APC-independent anti-inflammatory and cytoprotective functions of TM

The direct anti-inflammatory function of TM is preserved in its lectin-like domain (Table 1). The lectin-like domain of TM binds HMGB1 and inhibits its signaling via RAGE (Figure 2). UV irradiation-induced inflammation, in which HMGB1 plays a role, is alleviated by administration of the lectin-like domain of TM in association with a decrease in leukocyte infiltration and expression of TNFα as assessed by immunohistochemistry [63]. Of note, the use of the lectin-like domain of TM apparently improves the survival of LPS-challenged mice [63]. *In vivo* experiments with transgenic mice lacking the lectin-like domain of TM (TM^LeD/LeD^) also provide evidence for important anti-inflammatory roles of this domain; exposure of TM^LeD/LeD^ mice to LPS causes shorter survival in association with the infiltration of more polymorphonuclear leukocytes in organs, including lungs, compared to wild-type counterparts (TM^wt/wt^ mice) [64]. Further studies have found that the lectin-like domain of TM inhibits LPS-induced cytokine production and adhesion of neutrophils to endothelial cells in association with the suppression of ERK and NF-κB [64,65]. Curiously, this domain binds to LPS and gram-negative bacteria and induces their agglutination and enhances bacterial phagocytosis in macrophages [64]. Thus, the lectin-like domain of TM exerts its anti-inflammatory functions via various mechanisms.

Other targets of TM to alleviate inflammation include complement factors. Thrombin binds to TM efficiently and activates thrombin-activatable fibrinolysis inhibitor (TAFI), a procarboxypeptidase that hampers fibrinolysis by removing C-terminal lysine residues on fibrin that are otherwise important for the binding of plasminogen and t-PA, thereby efficiently generating plasmin [66,67]. The E3456 repeats of the EGF-like domain of TM are required to activate TAFI. The activated TAFI is capable of inactivating complement C3a and C5a (Table 1) [68,69]. In addition, based on the observation that TM^LeD/LeD^ mice lacking the lectin-like domain of TM are more susceptible to a mixture of monoclonal anti-collagen type II antibody-induced arthritis, in which synovial thickening and infiltration of inflammatory cells positive for compliment factors, including the membrane attack complex, are featured, it has been discovered that the lectin-like domain of TM interferes with complement activation via the classical and lectin pathways (Table 1) [70].

Similar to APC, TM also directly binds and inactivates histones [61,62]. Curiously, rTM inhibits extracellular histone-induced thrombus formation in pulmonary capillaries and subsequent right-sided heart failure [62]. The negatively charged domains of TM, the O-linked chondroitin sulfate glycosaminoglycan (GAG) moiety, are likely to interact with cationic proteins including histones. In fact, eosinophil-specific cationic protein, major basic protein, binds to TM via the GAG moiety and dampens its ability to generate APC, thereby promoting fibrin clot formation [71]. However, electrostatic interactions may not be the case for the binding complex formation of TM and histones; the binding affinity of TM lacking chondroitin sulfate is identical to that of TM containing chondroitin sulfate [61]. Thus, the sites in TM critical for binding to histones remain unknown [61,62].

The minimum structure of TM to generate cytoprotective activity is localized in the E45 repeats of the EGF-like domain (Table 1, Figure 2). The EGF-like domain of TM protects calcineurin inhibitor- or IL-1β-induced apoptosis in HUVECs in association with ERK-mediated upregulation of the antiapoptotic protein Mcl-1. Importantly, this effect is distinctive from that of APC, as single nucleotide substitutions at codons 376 or 424 of TM, which impair the ability of TM to produce APC or bind to thrombin, respectively, do not hamper the cytoprotective effects of TM [72].

## Conclusions

The TM/APC system, a guardian of blood coagulation and vascular integrity, is compromised in sepsis complicated by DIC. Therapeutic strategies to reconstitute the TM/APC system may mitigate inflammation and organ damage associated with inhibition of thrombus formation in septic DIC patients. These beneficial effects would cause improvement of the survival rate of this potentially lethal disease. The results of a phase III clinical trial of rTM are awaited to confirm the efficacy and safety of this agent in septic DIC patients.

## Abbreviations

TM: Thrombomodulin
APC: Activated protein C
rTM: Recombinant human soluble thrombomodulin
rhAPC: Recombinant human activated protein C
DIC: Disseminated intravascular coagulation
TF: Tissue factor
FVIIa: Activated factor VII
PAMPs: Pathogen-associated molecular patterns
DAMPs: Damage-associated molecular patterns
HMGB1: High-mobility group box 1
IL-6: Interleukin-6
TNFα: Tumor necrosis factor α
NETs: Neutrophil extracellular traps
PAI-1: Plasminogen activator inhibitor-1
AT: Antithrombin
TFPI: Tissue factor pathway inhibitor
PROWESS: Protein C Worldwide Evaluation in Severe Sepsis
BCSH: British Committee for Standards in Haematology
Gla-domain: Amino-terminal gamma-carboxyglutamic acid domain
EGF: Epidermal growth factor
EPCR: Endothelial cell protein C receptor
PAR1: Protease-activated receptor-1
S1P_1_: Sphingosine 1-phosphate receptor
Ang1: Angiopoietin 1
ALI: Acute lung injury
ARDS: Acute respiratory distress syndrome
PI3K: Phosphoinositide 3-kinase
ERK: Extracellular signal-regulated kinase
HUVECs: Human umbilical vein endothelial cells
NF-κB: Nuclear factor-κ B
TAFI: Thrombin-activatable fibrinolysis inhibitor
GAG: Glycosaminoglycan
ApoER2: Apolipoprotein E receptor 2
GSK3β: Glycogen synthase kinase 3β
LPS: Lipopolysaccharide
RGD: Arg-Gly-Asp

## References

[1] Gando S, Saitoh D, Ogura H, Mayumi T, Koseki K, Ikeda T, Ishikura H, Iba T, Ueyama M, Eguchi Y, Ohtomo Y, Okamoto K, Kushimoto S, Endo S, Shimazaki S, Japanese Association for Acute Medicine Disseminated Intravascular Coagulation (JAAM DIC) Study Group (2008). Natural history of disseminated intravascular coagulation diagnosed based on the newly established diagnostic criteria for critically ill patients: results of a multicenter, prospective survey. Crit Care Med.

[2] Ruf W, Edgington TS (1994). Structural biology of tissue factor, the initiator of thrombogenesis in vivo. FASEB J.

[3] Levi M, Keller TT, van Gorp E, ten Cate H (2003). Infection and inflammation and the coagulation system. Cardiovasc Res.

[4] Zeerleder S, Hack CE, Wuillemin WA (2005). Disseminated intravascular coagulation in sepsis. Chest.

[5] Semeraro N, Ammollo CT, Semeraro F, Colucci M (2012). Sepsis, thrombosis and organ dysfunction. Thromb Res.

[6] Ichinose A (2012). Factor XIII is a key molecule at the intersection of coagulation and fibrinolysis as well as inflammation and infection control. Int J Hematol.

[7] Harris HE, Raucci A (2006). Alarmin(g) news about danger: workshop on innate danger signals and HMGB1. EMBO Rep.

[8] Allam R, Kumar SV, Darisipudi MN, Anders HJ (2014). Extracellular histones in tissue injury and inflammation. J Mol Med.

[9] Zhang Q, Raoof M, Chen Y, Sumi Y, Sursal T, Junger W, Brohi K, Itagaki K, Hauser CJ (2010). Circulating mitochondrial DAMPs cause inflammatory responses to injury. Nature.

[10] Fiuza C, Bustin M, Talwar S, Tropea M, Gerstenberger E, Shelhamer JH, Suffredini AF (2003). Inflammation-promoting activity of HMGB1 on human microvascular endothelial cells. Blood.

[11] Xu J, Zhang X, Monestier M, Esmon NL, Esmon CT (2011). Extracellular histones are mediators of death through TLR2 and TLR4 in mouse fatal liver injury. J Immunol.

[12] Brinkmann V, Reichard U, Goosmann C, Fauler B, Uhlemann Y, Weiss DS, Weinrauch Y, Zychlinsky A (2004). Neutrophil extracellular traps kill bacteria. Science.

[13] Martinod K, Wagner DD (2014). Thrombosis: tangled up in NETs. Blood.

[14] Fuchs TA, Brill A, Duerschmied D, Schatzberg D, Monestier M, Myers DD, Wrobleski SK, Wakefield TW, Hartwig JH, Wagner DD (2010). Extracellular DNA traps promote thrombosis. Proc Natl Acad Sci USA.

[15] Jochum M, Lander S, Heimburger N, Fritz H (1981). Effect of human granulocytic elastase on isolated human antithrombin III. Hoppe Seylers Z Physiol Chem.

[16] Lin FY, Tsai YT, Lee CY, Lin CY, Lin YW, Li CY, Shih CM, Huang CY, Chang NC, Tsai JC, Chen TL, Tsai CS (2011). TNF-α-decreased thrombomodulin expression in monocytes is inhibited by propofol through regulation of tristetraprolin and human antigen R activities. Shock.

[17] Hack CE (2001). Fibrinolysis in disseminated intravascular coagulation. Semin Thromb Haemost.

[18] Suzuki K, Hayashi T, Nishioka J, Kosaka Y, Zushi M, Honda G, Yamamoto S (1989). A domain composed of epidermal growth factor-like structures of human thrombomodulin is essential for thrombin binding and for protein C activation. J Biol Chem.

[19] Ikezoe T (2014). Pathogenesis of disseminated intravascular coagulation in patients with acute promyelocytic leukemia, and its treatment using recombinant human soluble thrombomodulin. Int J Hematol.

[20] Shirahata A, Mimuro J, Takahashi H, Tsuji H, Kitajima I, Matsushita T, Eguchi Y, Kitamura N, Honda G, Sakata Y (2014). Postmarketing surveillance of recombinant human soluble thrombomodulin (thrombomodulin α) in pediatric patients with disseminated intravascular coagulation. Clin Appl Thromb Hemost.

[21] Mimuro J, Takahashi H, Kitajima I, Tsuji H, Eguchi Y, Matsushita T, Kuroda T, Sakata Y (2013). Impact of recombinant soluble thrombomodulin (thrombomodulin alfa) on disseminated intravascular coagulation. Thromb Res.

[22] Yamakawa K, Ogura H, Fujimi S, Morikawa M, Ogawa Y, Mohri T, Nakamori Y, Inoue Y, Kuwagata Y, Tanaka H, Hamasaki T, Shimazu T (2013). Recombinant human soluble thrombomodulin in sepsis-induced disseminated intravascular coagulation: a multicenter propensity score analysis. Intensive Care Med.

[23] Ikezoe T, Ikenoue N, Uchikawa N, Kojima S, Fukaya T, Yokoyama A (2010). Use of recombinant human soluble thrombomodulin in the management of HELLP syndrome complicated by DIC. Thromb Res.

[24] Sakai M, Ikezoe T, Bandobashi K, Yokoyama A (2010). Successful treatment of thrombotic thrombocytopenic purpura associated with systemic lupus erythematosus with recombinant human soluble thrombomodulin. Thromb Res.

[25] Sakai M, Ikezoe T, Bandobashi K, Togitani K, Yokoyama A (2010). Successful treatment of transplantation-associated thrombotic microangiopathy with recombinant human soluble thrombomodulin. Bone Marrow Transplant.

[26] Ikezoe T, Togitani K, Komatsu N, Isaka M, Yokoyama A (2010). Successful treatment of sinusoidal obstructive syndrome after hematopoietic stem cell transplantation with recombinant human soluble thrombomodulin. Bone Marrow Transplant.

[27] Ikezoe T, Takeuchi A, Taniguchi A, Togitani K, Yokoyama A (2011). Recombinant human soluble thrombomodulin counteracts capillary leakage associated with engraftment syndrome. Bone Marrow Transplant.

[28] Ikezoe T, Takeuchi A, Chi S, Takaoka M, Anabuki K, Kim T, Anabuki K, Sakai M, Taniguchi A, Togitani K, Yokoyama A (2013). Effect of recombinant human soluble thrombomodulin on clinical outcomes of patients with coagulopathy after hematopoietic stem cell transplantation. Eur J Haematol.

[29] Bernard GR, Vincent JL, Laterre PF, LaRosa SP, Dhainaut JF, Lopez-Rodriguez A, Steingrub JS, Garber GE, Helterbrand JD, Ely EW, Fisher CJ (2001). Recombinant human protein C Worldwide Evaluation in Severe Sepsis (PROWESS) study group: efficacy and safety of activated protein C for severe sepsis. N Engl J Med.

[30] Levi M, Toh CH, Thachil J, Watson HG (2009). Guidelines for the diagnosis and management of disseminated intravascular coagulation. British J Haematol.

[31] Thachil J, Toh CH, Levi M, Watson HG (2012). The withdrawal of activated protein C from the use in patients with severe sepsis and DIC [amendment to the BCSH guideline on disseminated intravascular coagulation]. Br J Haematol.

[32] Suzuki K, Kusumoto H, Deyashiki Y, Nishioka J, Maruyama I, Zushi M, Kawahara S, Honda G, Yamamoto S, Horiguchi S (1987). Structure and expression of human thrombomodulin, a thrombin receptor on endothelium acting as a cofactor for protein C activation. EMBO J.

[33] Dittman WA, Majerus PW (1990). Structure and function of thrombomodulin: a natural anticoagulant. Blood.

[34] Esmon CT, Esmon NL, Harris KW (1982). Complex formation between thrombin and thrombomodulin inhibits both thrombin-catalyzed fibrin formation and factor V activation. J Biol Chem.

[35] Dahlback B, Villoutreix BO (2005). The anticoagulation protein C pathway. FEBS Lett.

[36] Foster DC, Yoshitake S, Davie EW (1985). The nucleotide sequence of the gene for human protein C. Proc Natl Acad Sci USA.

[37] O'Brien LM, Mastri M, Fay PJ (2000). Regulation of factor VIIIa by human activated protein C and protein S: inactivation of cofactor in the intrinsic factor Xase. Blood.

[38] Kalafatis M, Rand MD, Mann KG (1994). The mechanism of inactivation of human factor V and human factor Va by activated protein C. J Biol Chem.

[39] Stearns DJ, Kurosawa S, Esmon CT (1989). Microthrombomodulin. Residues 310–486 from the epidermal growth factor precursor homology domain of thrombomodulin will accelerate protein C activation. J Biol Chem.

[40] Stearns-Kurosawa DJ, Kurosawa S, Mollica JS, Ferrell GL, Esmon CT (1996). The endothelial cell protein C receptor augments protein C activation by the thrombin-thrombomodulin complex. Proc Natl Acad Sci USA.

[41] Sakata Y, Curriden S, Lawrence D, Griffin JH, Loskutoff DJ (1985). Activated protein C stimulates the fibrinolytic activity of cultured endothelial cells and decreases antiactivator activity. Proc Natl Acad Sci USA.

[42] Sakamoto T, Ogawa H, Takazoe K, Yoshimura M, Shimomura H, Moriyama Y, Arai H, Okajima K (2003). Effect of activated protein C on plasma plasminogen activator inhibitor activity in patients with acute myocardial infarction treated with alteplase: comparison with unfractionated heparin. J Am Coll Cardiol.

[43] Schuepbach RA, Madon J, Ender M, Galli P, Riewald M (2012). Protease-activated receptor-1 cleaved at R46 mediates cytoprotective effects. J Thromb Haemost.

[44] Riewald M, Petrovan RJ, Donner A, Mueller BM, Ruf W (2002). Activation of endothelial cell protease activated receptor 1 by the protein C pathway. Science.

[45] Uchiba M, Okajima K, Oike Y, Ito Y, Fukudome K, Isobe H, Suda T (2004). Activated protein C induces endothelial cell proliferation by mitogen-activated protein kinase activation in vitro and angiogenesis in vivo. Circ Res.

[46] Finigan JH, Dudek SM, Singleton PA, Chiang ET, Jacobson JR, Camp SM, Ye SQ, Garcia JG (2005). Activated protein C mediates novel lung endothelial barrier enhancement: role of sphingosine 1-phosphate receptor transactivation. J Biol Chem.

[47] Bae JS, Kim YU, Park MK, Rezaie AR (2009). Concentration dependent dual effect of thrombin in endothelial cells via Par-1 and Pi3 kinase. J Cell Physiol.

[48] Minhas N, Xue M, Fukudome K, Jackson CJ (2010). Activated protein C utilizes the angiopoietin/Tie2 axis to promote endothelial barrier function. FASEB J.

[49] Bae JS, Rezaie AR (2010). Thrombin upregulates the angiopoietin-Tie2 axis: endothelial protein C receptor occupancy prevents the thrombin mobilization of angiopoietin 2 and P-selectin from Weibel-Palade bodies. J Thromb Haemost.

[50] Guo H, Liu D, Gelbard H, Cheng T, Insalaco R, Fernández JA, Griffin JH, Zlokovic BV (2004). Activated protein C prevents neuronal apoptosis via protease activated receptors 1 and 3. Neuron.

[51] Bir N, Lafargue M, Howard M, Goolaerts A, Roux J, Carles M, Cohen MJ, Iles KE, Fernández JA, Griffin JH, Pittet JF (2011). Cytoprotective-selective activated protein C attenuates *Pseudomonas aeruginosa*-induced lung injury in mice. Am J Respir Cell Mol Biol.

[52] Waerhaug K, Kuzkov VV, Kuklin VN, Mortensen R, Nordhus KC, Kirov MY, Bjertnaes LJ (2009). Inhaled aerosolised recombinant human activated protein C ameliorates endotoxin-induced lung injury in anaesthetised sheep. Crit Care.

[53] Maniatis NA, Letsiou E, Orfanos SE, Kardara M, Dimopoulou I, Nakos G, Lekka ME, Roussos C, Armaganidis A, Kotanidou A (2010). Inhaled activated protein C protects mice from ventilator-induced lung injury. Crit Care.

[54] Kerschen EJ, Fernandez JA, Cooley BC, Yang XV, Sood R, Mosnier LO, Castellino FJ, Mackman N, Griffin JH, Weiler H (2007). Endotoxemia and sepsis mortality reduction by non-anticoagulant activated protein C. J Exp Med.

[55] Geiger H, Pawar SA, Kerschen EJ, Nattamai KJ, Hernandez I, Liang HP, Fernández JÁ, Cancelas JA, Ryan MA, Kustikova O, Schambach A, Fu Q, Wang J, Fink LM, Petersen KU, Zhou D, Griffin JH, Baum C, Weiler H, Hauer-Jensen M (2012). Pharmacological targeting of the thrombomodulin-activated protein C pathway mitigates radiation toxicity. Nat Med.

[56] Yang XV, Banerjee Y, Fernández JA, Deguchi H, Xu X, Mosnier LO, Urbanus RT, de Groot PG, White-Adams TC, McCarty OJ, Griffin JH (2009). Activated protein C ligation of ApoER2 (LRP8) causes Dab1-dependent signaling in U937 cells. Proc Natl Acad Sci USA.

[57] Cao C, Gao Y, Li Y, Antalis TM, Castellino FJ, Zhang L (2010). The efficacy of activated protein C in murine endotoxemia is dependent on integrin CD11b. J Clin Invest.

[58] Elphick GF, Sarangi PP, Hyun YM, Hollenbaugh JA, Ayala A, Biffl WL, Chung HL, Rezaie AR, McGrath JL, Topham DJ, Reichner JS, Kim M (2009). Recombinant human activated protein C inhibits integrin-mediated neutrophil migration. Blood.

[59] Joyce DE, Gelbert L, Ciaccia A, DeHoff B, Grinnell BW (2001). Gene expression profile of antithrombotic protein c defines new mechanisms modulating inflammation and apoptosis. J Biol Chem.

[60] Xu J, Zhang X, Pelayo R, Monestier M, Ammollo CT, Semeraro F, Taylor FB, Esmon NL, Lupu F, Esmon CT (2009). Extracellular histones are major mediators of death in sepsis. Nat Med.

[61] Ammollo CT, Semeraro F, Xu J, Esmon NL, Esmon CT (2011). Extracellular histones increase plasma thrombin generation by impairing thrombomodulin-dependent protein C activation. J Thromb Haemost.

[62] Nakahara M, Ito T, Kawahara K, Yamamoto M, Nagasato T, Shrestha B, Yamada S, Miyauchi T, Higuchi K, Takenaka T, Yasuda T, Matsunaga A, Kakihana Y, Hashiguchi T, Kanmura Y, Maruyama I (2013). Recombinant thrombomodulin protects mice against histone-induced lethal thromboembolism. PLoS One.

[63] Abeyama K, Stern DM, Ito Y, Kawahara K, Yoshimoto Y, Tanaka M, Uchimura T, Ida N, Yamazaki Y, Yamada S, Yamamoto Y, Yamamoto H, Iino S, Taniguchi N, Maruyama I (2005). The N-terminal domain of thrombomodulin sequesters high-mobility group-B1 protein, a novel antiinflammatory mechanism. J Clin Invest.

[64] Shi CS, Shi GY, Hsiao HM, Kao YC, Kuo KL, Ma CY, Kuo CH, Chang BI, Chang CF, Lin CH, Wong CH, Wu HL (2008). Lectin-like domain of thrombomodulin binds to its specific ligand Lewis Y antigen and neutralizes lipopolysaccharide-induced inflammatory response. Blood.

[65] Conway EM, Van de Wouwer M, Pollefeyt S, Jurk K, Van Aken H, De Vriese A, Weitz JI, Weiler H, Hellings PW, Schaeffer P, Herbert JM, Collen D, Theilmeier G (2002). The lectin-like domain of thrombomodulin confers protection from neutrophil-mediated tissue damage by suppressing adhesion molecule expression via nuclear factor kappaB and mitogen-activated protein kinase pathways. J Exp Med.

[66] Bajzar L, Manuel R, Nesheim ME (1995). Purification and characterization of TAFI, a thrombin-activable fibrinolysis inhibitor. J Biol Chem.

[67] Wang W, Boffa MB, Bajzar L, Walker JB, Nesheim ME (1998). A study of the mechanism of inhibition of fibrinolysis by activated thrombin-activable fibrinolysis inhibitor. J Biol Chem.

[68] Campbell WD, Lazoura E, Okada N, Okada H (2002). Inactivation of C3a and C5a octapeptides by carboxypeptidase R and carboxypeptidase N. Microbiol Immunol.

[69] Leung LL, Myles T, Nishimura T, Song JJ, Robinson WH (2008). Regulation of tissue inflammation by thrombin-activatable carboxypeptidase B (or TAFI). Mol Immunol.

[70] Wang H, Vinnikov I, Shahzad K, Bock F, Ranjan S, Wolter J, Kashif M, Oh J, Bierhaus A, Nawroth P, Kirschfink M, Conway EM, Madhusudhan T, Isermann B (2012). The lectin-like domain of thrombomodulin ameliorates diabetic glomerulopathy via complement inhibition. Thromb Haemost.

[71] Slungaard A, Vercellotti GM, Tran T, Gleich GJ, Key NS (1993). Eosinophil cationic granule proteins impair thrombomodulin function. A potential mechanism for thromboembolism in hypereosinophilic heart disease. J Clin Invest.

[72] Ikezoe T, Yang J, Nishioka C, Honda G, Furihata M, Yokoyama A (2012). Thrombomodulin protects endothelial cells from a calcineurin inhibitor-induced cytotoxicity by upregulation of extracellular signal-regulated kinase/myeloid leukemia cell-1 signaling. Arterioscler Thromb Vasc Biol.

